# Engaging Primary Care Physicians to Refer Patients to Home-Based Palliative Is Challenging and Complicated

**DOI:** 10.1089/pmr.2020.0009

**Published:** 2020-11-05

**Authors:** Alexis Coulourides Kogan, Kelly Sadamitsu, Michael Gaddini, Michael Kersten, Jeanine Ellinwood, Torrie Fields

**Affiliations:** ^1^ Department of Family Medicine and Geriatrics, Keck School of Medicine of USC, University of Southern California, Alhambra, California, USA.; ^2^ Snowline, Sacramento, California, USA.; ^3^ Hill Physicians Medical Group, Sacramento, California, USA.; ^4^ Blue Shield of California, San Francisco, California, USA.

**Keywords:** home-based palliative care, physician engagement, primary care, qualitative methods, serious illness

## Abstract

***Background:*** Before the Affordable Care Act (ACA), the financing landscape for fee-for-service health care lacked broad structure and incentives to provide palliative care outside hospitals. Since the ACA, several payers have taken the opportunity to offer home-based palliative care (HBPC) to their members.

***Objective:*** To evaluate the impact of outreach efforts by a physician champion among a cohort of primary care physicians (PCPs) to introduce a new HBPC program and benefit, obtain buy-in, and motivate referrals for Blue Shield patients.

***Design:*** Secondary qualitative analysis of detailed field notes from a HBPC physician champion from in-person meetings with a cohort of PCPs and their office staff.

***Subjects:*** PCPs were from a physicians group in northern California that met with the physician champion during a 12-month study period.

***Results:*** During the 12-month study period, the physician champion met with clinicians at 27 distinct primary care offices. Qualitative analyses revealed three independent themes relating to receptivity and perception of the new HBPC program: (1) physician-level factors (overburdened, lack of palliative care knowledge, misconceptions around palliative care, and patient control), (2) practice-level factors (practice structure and role/integration of advance practice providers), and (3) first impression of the HBPC program (receptivity, “dirty data,” and communication).

***Conclusion:*** Results hold important implications for practice and new approaches to engaging PCPs in HBPC, obtaining buy-in, and generating patient referrals. PCPs need better support in caring for patients with serious illness and HBPC can likely fill that role if PCPs are willing to refer and HBPC programs adapt.

Before the Affordable Care Act (ACA), the financing landscape for fee-for-service health care lacked broad structure and incentives to provide palliative care—medical care focused on providing pain and symptom relief for seriously ill patients, with the goal of improving quality of life^1,2^—outside hospitals. Since the ACA, several payers have taken the opportunity to offer home-based palliative care (HBPC) to their members. We evaluated the impact of outreach efforts by a physician champion among a cohort of primary care physicians (PCPs) to introduce a new HBPC program and benefit, obtain buy-in, and motivate referrals for Blue Shield patient members. With Institutional Review Board approval, we conducted a secondary qualitative analysis of bi-monthly detailed field notes by a single HBPC physician champion from in-person meetings with a cohort of PCPs and their office staff, of which Blue Shield members comprised about a third of their patients. Participants were PCPs from a single physicians group in northern California who met with the physician champion during a 12-month study period. Field notes were analyzed independently, then jointly, by two researchers using a grounded theory approach^3,4^ to identify and code themes that arose from the data.

During the study period, the physician champion met with clinicians at 27 distinct primary care offices. On average, PCPs were contacted two to three times (beyond the initial scheduling/rescheduling; range: 1–5, $\bar{x}$ = 2.63 ± 1.04), which consisted of an in-person meeting with the physician champion (73.0%), e-mail contact (13.1%), telephone call (7.0%), or in-person visit with staff/advance practice providers (APPs) (7.0%). At the end of the study period, five patients were referred to the HBPC program: all coming from one physician from a group practice (Table 1).

**Table 1.** Participant Characteristics

	Solo practice	Group practice	Overall
	n = 18	n = 9	n = 27
Specialty			
Family medicine	7	5	12
Internal medicine	11	4	15
Patients cared for			
Overall range	210–1148	161–5639	446–1312
Mean (SD)	757 (379.5)	1810 (1565.2)	1108 (1050.5)
BSC range	67–679	87–1697	111–679
BSC mean (SD)	224 (139.7)	581 (465.5)	356 (325.0)
Percent BSC (SD)	32 (7.1)	35 (8.1)	33 (7.4)
Provider count (%)			
1	18 (100)	0 (0)	18 (66.7)
2	—	3 (33.2)	3 (11.1)
3	—	4 (44.4)	4 (14.8)
4	—	2 (22.2)	2 (7.4)
Patient referrals made	0 (0)	5 (100.0)	—

BSC, Blue Shield of California; SD, standard deviation.

Qualitative analyses of the physician champion's field notes revealed three independent themes relating to receptivity and perception of the new HBPC program: (1) physician-level factors (lack of time, lack of palliative care health literacy, and a desire to retain oversight of their patients' care), (2) practice-level factors (differences in solo versus group practice structures and the integration of APPs), and (3) first impression of the HBPC program (receptivity, “dirty” claims data, and communication) (Table 2).

**Table 2.** Qualitative Results

Theme	Subtheme	Note ID number	Quotation from field notes
1. Physician-level factors			
	1.a. Overburdened/info not retained	106	“The other thing that is obvious is that physicians in practice are quite overwhelmed and [it] is difficult for them to comprehend the specifics of this program. I think it is important…That we walk through exactly the steps that happen…so the physicians understand how our services will be integrated with their care of their patients”
		109	“What was remarkable to me was that neither he nor his nurse practitioner seem to have any memory of the program from our presentation of it late in the summer”
		110	“Even though you present the program competently to a practitioner and it is well-received does not mean that they retain knowledge of it and put it to use”
	1.b. Lack of palliative care knowledge	116	“We spent a great deal of time talking about the differences between palliative care and hospice, and that code status really had nothing to do with the patient qualifying”
		111	“…He felt that we were trying to force hospice on his patients…The discussion mostly reflected on his bias…as he mentioned that hospice is useful when a patient has ‘weeks to live’”
	1.c. Misconceptions about palliative care	116	“He stated emphatically that if his patients [end] up going to the emergency room, that this program would not work for him…I think the PCP's expectations are entirely unrealistic and he is focusing on the fact that his patient ended up being hospitalized”
	1.d. Patient control	114	“He got very upset and accused us of trolling for patients”
		115	“He prefers to be contacted personally to give a personal okay before referrals are done…that [doing otherwise] represented ‘heavy handling’ by the insurance”
2. Practice-level factors			
	2.a. Practice structure	110	“We also discussed the difference between working with institutional physicians and employed physicians versus self-employed physicians. We have a much harder job of promoting the program [with the latter]”
		106	“…Especially for the self-employed physicians…[we need to] develop strategies to address confusions [sic]… how our program works along with them and complements them. We need to integrate this program with their care and make sure that the physicians don't see these two [as] competing”
	2.b. APPs	110	“We also need to reach out more to mid-levels [PAs, NPs] as I think their retention and use of the program is higher”
		109	“We were unable to meet with his mid-level [NP] but I thought that if we were a [sic] we might be able to generate some more use of the program from the office”
3. First impression of the HBPC program			
	3.a. Receptivity	109	“Again, I am amazed at the variation in receptivity in our program as some physicians wholeheartedly embrace it while others seem cautious”
		108	“I think once he got familiar with the program, he would use it much more readily”
	3.b. Dirty data	113	“He received his latest list and reported that 12 out of 13 patients were either dead or not at all entirely appropriate…This reinforces that the lists of patients going to physicians are not adequately identifying the patients and [that this] may detract from our efforts to promote this program”
		103	“All patients [on the list]…were hospitalized for injuries or serious illnesses, but they tended to be younger, and none of them [chronically] sick”
	3.c. Communication	108	“I think it would be useful to have a variable approach in working with MD's from consultative to collaborative. Fewer docs seem willing for us to independently manage the patients until they know and trust us”
		107	“He didn't seem eager to refer and voiced concerns that folks would come in and change a bunch of meds on his patients without consulting him first. He definitely favored a more consultative approached and stressed the importance of regular communication”
		109	“When I gave him the option of how involved we wanted to be with his patients he remarked that he would like us to take over care completely”

APPs, advance practice providers; HBPC, home-based palliative care; NP, nurse practitioner; PA, physician assistant; PCPs, primary care physicians.

Several of the physician-level factors identified are corroborated by previous research that has also found time constraints, lack of palliative care health literacy, and misconceptions of palliative care among primary care and specialty physicians to impede referral to palliative care.^5–10^ Another study found that even among PCPs who held positive beliefs about palliative care, that was still not enough to motivate patient referral.^11^ These findings are concerning as they relate to low patient referral to palliative care but they also highlight a difficult reality for physicians: that even a positive attitude may not be enough to promote referral if PCPs are constrained for time. However, these findings still hold important implications for future proliferation and success of new HBPC programs (something that influential groups have called for^1,2,12,13^) by threatening their sustainability.

On a positive note, in contrast to our findings, several factors *have* been found in previous research to be associated with patient referral to palliative care including high program visibility,^11^ personal experience with palliative care^11^ or advance care planning,^5^ and longer tenure at respective organization/practice.^5,11^ In addition, other recommended strategies for engaging physicians in quality improvement, also applicable to palliative care, are engaging leadership, utilizing a physician champion, conducting physician outreach, providing physician education, tailoring program to match style/preference of physician, and ensuring innovations-values fit with the program and physicians.^7,14^ Several of these strategies were already implemented in the palliative care program in this study and others such as refining the educational, marketing, and outreach materials and language used among the PCPs could be tried and evaluated in a subsequent study They may also suggest key actions that brand new HBPC programs can take in an effort to engage PCPs more fully and other clinicians who may refer patients to the service. Further research is warranted.

Another key finding was the full spectrum of PCP willingness to either give up or retain full control of their palliative-eligible patients. With a few exceptions, most PCPs preferred maintaining full oversight of their patients, and this was a major factor contributing to their unwillingness to refer to the HBPC program. Relatedly, some PCPs reported feeling upset when their patient was identified or referred to HBPC by someone else (i.e., medical group case managers or payer lists of preidentified patients presented by the physician champion), especially when that source had ties to the payer, specifying that it felt like an inappropriate overreach and potential phishing scheme. These perceptions, however real or perceived, are important to acknowledge and address from the PCP's point of view, especially since private practice in particular is often the target for companies selling in the health care sector and may raise suspicion.^15^ Recent research recognizes these concerns among PCPs and suggests that they may be exacerbated by the new yet growing business sector of palliative care coupled with nebulous aspects of HBPC such as lack of a single model of care, various payment models, and variable patient eligibility.^16^ A lot of work is needed in this area,^16^ but HBPC programs may focus their efforts on improving PCP palliative care health literacy, transparency, and communication with PCPs to begin to effect change now.

One recommended strategy to overcome PCP variance in willingness or resistance to maintaining control of their patients' care is for the palliative care team to tailor their communication, consultation, and collaboration with the PCP to match the level desired by the PCP.^14^ Although it would require effort to sort out styles and preferences, the potential to establish and grow trust, confidence, and collaboration between the PCP and HBPC team could be extremely valuable for all partners in care. In addition, this approach may touch on another important notion from study findings: the idea of “just try one” patient (a subsequent experience the HBPC program has control over). If PCPs have a positive experience mutually caring for a patient with the HBPC team in the PCPs' preferred collaboration style, they may develop a deeper understanding for and a better-informed opinion of the service. Further investigation in this area is needed to understand more fully the effects of HBPC tailored to PCP preferences; however, a first impression may go a long way. For example, during the meetings with the physician champion, several physicians described “good” and “bad” first impressions of the HBPC program. One physician was pleased that two of their patients were identified and referred to HBPC by case managers from the medical group and that the HBPC team was updating the PCP along the way, facilitating care at home, and even obtained medical equipment. Conversely, another physician was angered by their patient being enrolled in HBPC by medical group case managers postdischarge from hospital where the PCP only learned about this when the HBPC team phoned the office requesting medical records (prior faxes had gone unnoticed). These experiences have significant implications for HBPC programs wherein each of these physicians likely has a very divergent view of the program, and thus, very divergent referral behavior. Since “first impression” is a factor impacting PCP referral that HBPC programs have the most control over, it is important for them to be communicative, transparent, and collaborative with PCPs. Results from this study corroborate the role of burden among PCPs,^17,18^ which was evidenced in several ways: high difficulty scheduling/rescheduling in-person contact with PCPs, PCPs juggling patient visits in between meeting with the physician champion, and comments from PCPs. High PCP burden may lead to feeling emotionally exhausted, which is one of three dimensions of burnout^19^: a costly syndrome characterized by detachment from work, perception of low personal achievement, medical errors, and poor patient outcomes.^19^ Previous research has documented difficulty in engaging physicians in innovations, such as new programs or models of care, when they are experiencing burnout.^14,18^ One potential strategy identified in this study and previous research to alleviate some burden on PCPs may be incorporation of APPs in primary care.^9,14^ This may be a possible solution for some practices but not suitable for all and could even create additional strain especially for solo practices to employ new personnel. Another strategy could be referring patients with complex care and social support needs to programs such as HBPC when possible and available. However, as this study found, that is exactly the predicament that many PCPs are faced with: being overburdened and short on time yet still unwilling to refer patients to a service despite its potential to alleviate some constraints.

Despite identifying important factors that precluded PCPs from referring patients to a new HBPC program, this study may be limited in several ways. First, it was conducted retrospectively and analyzed field notes from PCP encounters that were not originally intended for research purposes. This may have impacted the accuracy and robustness of the data since meetings were neither audio taped and transcribed nor followed a semistructured research protocol. Having a single physician champion conduct the meetings with an established discourse may have helped to limit these issues of the lack of a formalized research protocol and activities; however, it may also impede generalizability of findings. Relatedly, the single physician champion's approach and engagement/communication style could have influenced participants' responses. Second, participants were from a single physicians group in northern California. This may also limit the generalizability of study findings.

This study holds several important implications for practice and new approaches to engaging PCPs in HBPC, obtaining buy-in, and generating patient referrals. Findings suggest the crucial role that factors such as physician palliative care knowledge, time constraints, practice structure, and collaborative care styles and preferences serve to successfully engage PCPs in new models of care such as HBPC. It also brings into question the amount of information that PCPs can reasonably retain over a one-hour conversation or lunch meeting, which bears resemblance to in-office visits from pharmaceutical representatives. Several areas identified in this research may be amenable to change, however, it is likely that the largest barrier is lack of palliative care health literacy as it relates to the widespread clinician-held myth of equating palliative care with hospice.^1,5,8,9,11,20–22^ Correcting misconceptions such as this one may help PCPs begin to become more willing to try HBPC for some patients, thereby better meeting the multifaceted health and social needs of complex patients and reducing burden on themselves and their medical practice.

Running a primary care office is complex, especially for solo practices where the physician is engaged in both patient care and business operations. PCPs are already performing elements of palliative care, such as advance care planning,^23^ yet several key attributes of care for patients with serious illness are hard for PCPs to provide, such as emotional support, spiritual support, practical assistance, and social services.^6,24^ Considering that other specialists believe referrals to palliative care should come from PCPs,^8^ PCPs need better support in caring for their patients with serious illness, including identifying when specialized care is needed. HBPC can likely fill that role, if PCPs are willing to try and programs are willing to adapt.

## Abbreviations Used

ACA: Affordable Care Act
APPs: advance practice providers
HBPC: home-based palliative care
PCPs: primary care physicians
SD: standard deviation
